# Clinicopathological study of hepatic mesenchymal hamartoma and undifferentiated embryonal sarcoma of the liver: a single center study from Iran

**DOI:** 10.1186/s13000-021-01117-z

**Published:** 2021-06-24

**Authors:** Parham Habibzadeh, Mohaddese Ansari Asl, Hamid Reza Foroutan, Ali Bahador, Mohammad Hossein Anbardar

**Affiliations:** 1 Persian BayanGene Research and Training Center, Shiraz University of Medical Sciences, Shiraz, Iran; 2 Student Research Committee, Shiraz University of Medical Sciences, Shiraz, Iran; 3 Laparoscopy Research Center, Shiraz University of Medical Sciences, Shiraz, Iran; 4 Department of Pediatric Surgery, Nemazee Hospital, Shiraz University of Medical Sciences, Shiraz, Iran; 5 Department of Pathology, Shiraz Medical School, Shiraz University of Medical Sciences, Shiraz, Iran

**Keywords:** Undifferentiated embryonal sarcoma of the liver, Hepatic mesenchymal hamartoma, Liver neoplasms, Immunohistochemistry, Pathology, surgical, CD56 antigen, Sarcoma, Pediatric liver tumors

## Abstract

**Background:**

Undifferentiated embryonal sarcoma of liver (UESL) and hepatic mesenchymal hamartoma (HMH) are two rare entities which mainly affect the pediatric population. The aim of this investigation was to provide a comprehensive overview of the clinicopathologic characteristics of the patients diagnosed with these two conditions in a tertiary referral center in Iran.

**Methods:**

In this retrospective study patients diagnosed with UESL or HMH between 2012 and 2020 were studied. A comprehensive histopathologic evaluation of the cases along with immunohistochemistry evaluation using a panel of antibodies was conducted. Furthermore, clinical, paraclinical, and treatment data and follow up information was collected.

**Results:**

A total of 16 patients (8 UESL, 8 HMH) were studied in this investigation. Patients with UESL had a significantly (*p* = 0.002) higher age at diagnosis compared with those with HMH. Histologically, UESL cases were characterized by anaplastic cells with eosinophilic cytoplasm and bizarre nuclei and frequent atypical mitosis and spindling in a myxoid stroma while disordered arrangement of hepatic parenchyma, bile ducts, and primitive mesenchyme was seen in HMH. Furthermore, small round cells and extramedullary hematopoiesis were seen in 2 UESL and 3 HMH cases, respectively. Concurrent HMH was also seen in two UESL cases. Immunohistochemistry panel showed positive staining for Vimentin, Glypican-3, Desmin, CD56, CD10, and BCL2 in UESL cases and immunoreactivity for Vimentin, HepPar 1, Glypican-3, SMA, CD56, BCL2, and CD34 in various components of HMH.

**Conclusions:**

In this study, the clinicopathologic features of UESL and HMH cases are presented. We also evaluated the utility of an immunohistochemistry panel in the diagnosis of these two rare entities and suggested novel markers. Our study corroborated the findings of previous investigations and expanded the clinicopathologic features of these two rare entities with diagnostic and potential therapeutic implications.

## Background

Undifferentiated embryonal sarcoma of the liver (UESL) and hepatic mesenchymal hamartoma (HMH) are two rare pathologic entities that are primarily seen in the pediatric population [1]. UESL is a rare mesenchymal tumor accounting for 5–15% of liver malignancies in pediatrics [2–5]. On the other hand, HMH, accounting for 8% of liver tumors in children, comprises the majority of pediatric benign liver tumors after infantile hemangioma [1, 6]. UESL which is an aggressive tumor was first described in 1978 by Stocker and Ishak and is primarily diagnosed between 6 and 10 years of age without gender predominance [7, 8]. However, HMH is mainly diagnosed in children of less than 2 years of age and shows a slight male predominance in this age group [9]. UESL usually arises from the right hepatic lobe with tumor size varying from 10 cm to 30 cm [5]. Similarly, HMH is primarily seen in the right hepatic lobe in children and can have various sizes of up to 30 cm in diameter [9, 10]. Patients with UESL usually present with non-specific symptoms including anorexia, abdominal pain, fever, and nausea with subsequent findings of cystic and solid components in imaging studies [5, 7, 11, 12]. Abdominal distention or mass is the most common clinical presentation of HMH which is usually seen as a multi-loculated cyst with a varying solid component on radiologic studies [10].

The underlying pathologic mechanisms playing a role in the development of UESL and HMH are unclear. However, different studies have proposed a number of potential mechanisms. Comparative genome hybridization (CGH) studies of UESL have shown different patterns of chromosomal changes including losses of chromosome 9p, 11p, and 14 and gains of chromosome 1q, 5p, 6q, 8p, and 12q pointing to the potential role of chromosomal instability [13]. Genetic alterations leading to the ectopic activation of chromosome 19q microRNA cluster (C19MC) are found in HMH [14].

UESL is usually diagnosed based on the patient’s age, tumor location, and an immunohistochemistry panel of undifferentiated markers including vimentin, desmin, α_1_ anti trypsin, CD10, and CD68 [15, 16]. However, HMH is usually diagnosed using clinical and histopathologic features alone [1]. Overall, as a result of the low incidence of these pathologic entities, the clinicopathological features of UESL and MH are limited to case series and case reports. Considering the paucity of reports from the Middle Eastern region, this study was conducted to investigate the clinical, histological, and immunohistochemical features in a series of patients with UESL and HMH in a single referral center from Iran.

## Materials and methods

In this investigation, a retrospective evaluation of patients diagnosed with UESL and HMH at Shiraz University of Medical Sciences between 2012 and 2020 was conducted. The diagnosis was based on histopathological evaluation of tumor samples according to WHO classification of tumors of the digestive system. The specimens were obtained by surgical resection and were subsequently fixed in formalin and then embedded in paraffin. They were then stained using hematoxylin and eosin staining, periodic acid–Schiff (PAS) staining for UESL cases, and immunohistochemistical staining for all cases using the following antibodies: Vimentin (Máster Diagnóstica, rabbit monoclonal antibody, Clone SP20), HepPar 1 (Máster Diagnóstica, mouse monoclonal antibody, clone OCH1E5), Glypican 3 (Máster Diagnóstica, mouse monoclonal antibody, clone 1G12), Arginase-1 (Biocare Medical, rabbit monoclonal antibody, clone EP261), Ki67 (Máster Diagnóstica, rabbit monoclonal antibody, clone SP6), Desmin (Máster Diagnóstica, mouse monoclonal antibody, clone D33), SMA (Máster Diagnóstica, mouse monoclonal antibody, clone 1A4), CD56 (Máster Diagnóstica, rabbit monoclonal antibody, clone MRQ-42), CD10 (Máster Diagnóstica, mouse monoclonal antibody, clone 56C6), CD68 (Máster Diagnóstica, mouse monoclonal antibody, clone KP-1), BCL2 (Máster Diagnóstica, rabbit monoclonal antibody, clone EP36), PD-L1 (Máster Diagnóstica, rabbit monoclonal antibody, clone CAL10), C-Kit (Máster Diagnóstica, rabbit monoclonal antibody, clone EP10), CD34 (Máster Diagnóstica, mouse monoclonal antibody, clone QBEnd/10). Appropriate positive and negative controls were used throughout the experiments. The immunohistochemistry slides were subsequently evaluated by a pathologist. In the event that less than 1% of the cells in a slide showed immunoreactivity, the case was considered negative. The positive cases were subsequently graded based on staining intensity as weak, intermediate, and strong. If more than half of the cells of interest were stained, the staining was considered diffuse. Otherwise, in cases with a staining percentage between 1 and 50%, the staining was considered focal.

The following information was collected for both groups of the patients: patient age, sex, presenting symptoms, tumor location, significant laboratory findings. Furthermore, for patients diagnosed with UESL extrahepatic metastasis, recurrence, disease stage, and radiologic findings were collected. The treatments received by the patients with UESL and the disease outcome were also collected by contacting the family members. In addition, the gross and microscopic pathologic findings were documented for all the cases.

*R* ver 4.0.2 (2020-06-22) was used for statistical analysis. Considering the small sample size of the study, continuous variables were reported as the median and interquartile range (IQR). Mann-Whitney U test was used to compare the distribution of variables between two groups. The correlation between tumor size and age was assessed with Spearman’s *ρ*. Kaplan-Meier survival analysis for the patients with UESL. A *p* value < 0.05 was considered statistically significant.

## Results

The clinicopathologic characteristics and demographic data of the patients with UESL and HMH are presented in Tables 1 and 2, respectively.

**Table 1:** Clinical and demographic characteristics of patients with UESL. GTR: Gross tumor resection

Patient	Age	Sex	Tumor Location	Extrahepatic Metastasis	Symptoms	COG Stage	Laboratory Findings	CT Imaging Findings	Surgery	Radiation Therapy	Chemotherapy	Recurrence	Outcome (years from diagnosis
Case 1	2y 8 M	M	Right lobe	–	Abdominal pain, Fever	I	↑ AST, ALT	Lobulated hypodense mass with peripheral irregularity and focal calcification	GTR	–	+ (Vincristine, Actinomycin, Cyclophosphamide, Doxorubicin, Ifosfamide, Etoposide)	–	No evidence of disease (8y)
Case 2	14y	F	Right lobe	–	Abdominal pain, anorexia, weight loss	I	↑Plt, ↓Hb	Heterogeneous lesion with intratumoral hemorrhage and multiple foci of necrosis	GTR	–	+	–	No evidence of disease (6y)
Case 3	13y	F	Right lobe	–	Abdominal pain, nausea/vomiting	I	↑CA-125	Hypodense lesion with faint peripheral and internal septation	GTR	–	–	–	No evidence of disease (6y)
Case 4	12y	F	Right lobe	–	Abdominal pain	III	↓Hb	Cystic mass with solid nodules and septation	GTR	+	+	+	Died of disease (3y)
Case 5	3y 6 M	M	Right lobe	–	Abdominal pain	I	↑INR, ↓ Hb	Complicated hypoattenuated mass containing septation	GTR	–	+ (Actinomycin, Cyclophosphamide, Vincristine)	–	No evidence of disease (3.5y)
Case 6	12 y	F	Right lobe	–	Abdominal pain, nausea/vomiting	I	↑ AST, ALT	Hypoattenuating lesion with multiple thick septa	GTR	+	+	+	Under treatment (3y)
Case 7	5 y	M	Right lobe	–	Abdominal pain, nausea/vomiting	I	↑ AST, ALT	Cystic mass with internal septation	GTR	–	+ (Vincristine, Doxorubicin, Cyclophosphamide)	–	No evidence of disease (2y)
Case 8	16 y	F	Right lobe	–	Abdominal pain, chills, fever	II	↑INR, ↑ ESR, ↓ Hb	Heterogenous hypodense mass	GTR	–	+	–	No evidence of disease (1y)

**Table 2:** Clinicopathologic characteristics and demographic features of patients with HMH

Patient	Age (y)	Sex	Symptoms	Size (cm)	Location	Focality	Solid/Cystic	Extramedullary Hematopoiesis	Other Findings
Case 1	2.5	M	Abdominal Distention	15	Right Lobe	Unifocal	Solid	–	
Case 2	2.5	F	Abdominal Distention	12	Right Lobe	Unifocal	Cystic	–	
Case 3	1	F	Abdominal Mass	9.5	Right Lobe	Unifocal	Solid	–	
Case 4	1.5	M	Abdominal Mass/Diarrhea	12	Right Lobe	Unifocal	Solid	+	
Case 5	0.5	M	Abdominal Distention	17	Right Lobe	Unifocal	Solid	+	Hemorrhage/Severe Hepatic Steatosis/ Elevated AFP (1125 ng/ml)
Case 6	2.5	M	Abdominal Mass	14	Right Lobe	Unifocal	Solid	+	
Case 7	5	M	Abdominal Pain/Fever	8	Right Lobe	Unifocal	Cystic	–	
Case 8	2	F	Abdominal Distention	20	Right Lobe	Unifocal	Cystic	–	

Overall, there were 8 patients (3 males and 5 females) with UESL with a median age at diagnosis of 12.0 (IQR 4.6 to 13.3) years. In addition, 8 patients (5 males and 3 females) with HMH with a median age at diagnosis of 2.3 (IQR 1.4 to 2.5) years were identified. The age at diagnosis for those with UESL was significantly (*p* = 0.002) higher than those with HMH. Abdominal pain was the most common (8/8) presenting symptom in patients with UESL, followed by nausea/vomiting (3/8) and fever (2/8). Abdominal distention (4/8) and accidental identification of an abdominal mass by the patient’s caregiver (3/8) were the most common complaints on initial presentation in patients with HMH. All the UESL and HMH patients identified had right liver lobe masses. Except for one patient with UESL, all of the UESL and HMH masses identified were unifocal. Initial laboratory abnormalities were more commonly observed in patients with UESL compared with those with MH. Anemia (4/8) and elevated hepatic transaminases levels (3/8) were seen in patients with UESL. Notably, elevated CA-125 levels were seen in one of the patients with UESL. Furthermore, one patient with HMH had elevated alpha-fetoprotein (AFP) levels. All patients diagnosed with HMH had undergone surgical resection of the liver mass and were alive after a median follow-up of 5.5 years without any complications. Most of the patients with UESL underwent gross tumor resection followed by adjuvant chemotherapy (7/8). Furthermore, 2/8 patients underwent radiation therapy as well. In patients with UESL, after a median follow-up time of 3 years, one patient had passed away and one of the patients was still under treatment due to recurrence. Overall, tumor recurrence was observed in two of the patients. No evidence of the disease after the treatment was detected among the other six patients with UESL. Patients with UESL had a mean survival time of 7.2 (95% CI 5.7 to 8.7) years estimated by Kaplan-Meier survival analysis.

On gross examination, UESL was typically found as a well-defined mass with necrosis and hemorrhage. Gelatinous material in cases with myxoid change was also seen. The tumor size varied from 5.0 cm to 28.0 cm (median: 15.0 cm, IQR 10.0 cm to 19.0 cm) (Table 3).

**Table 3:** Findings on gross examination of UESL cases

Patient	Tumor Size (cm)	Focality	Well-defined/ill-defined	Cystic change	Hemorrhage	Necrosis	Myxoid Change
Case 1	10	Multifocal	Well-defined	–	+	+ (40%)	+
Case 2	18	Unifocal	Well-defined	+	+	+ (30%)	+
Case 3	28	Unifocal	Well-defined	+	+	+ (90%)	+
Case 4	10	Unifocal	Well-defined	+	+	+ (60%)	+
Case 5	14	Unifocal	Well-defined	–	+	+ (80%)	+
Case 6	22	Unifocal	Well-defined	+	+	+ (40%)	+
Case 7	5	Unifocal	Well-defined	+	+	+ (80%)	–
Case 8	16	Unifocal	Well-defined	+	+	+ (80%)	+

**Fig. 1:**
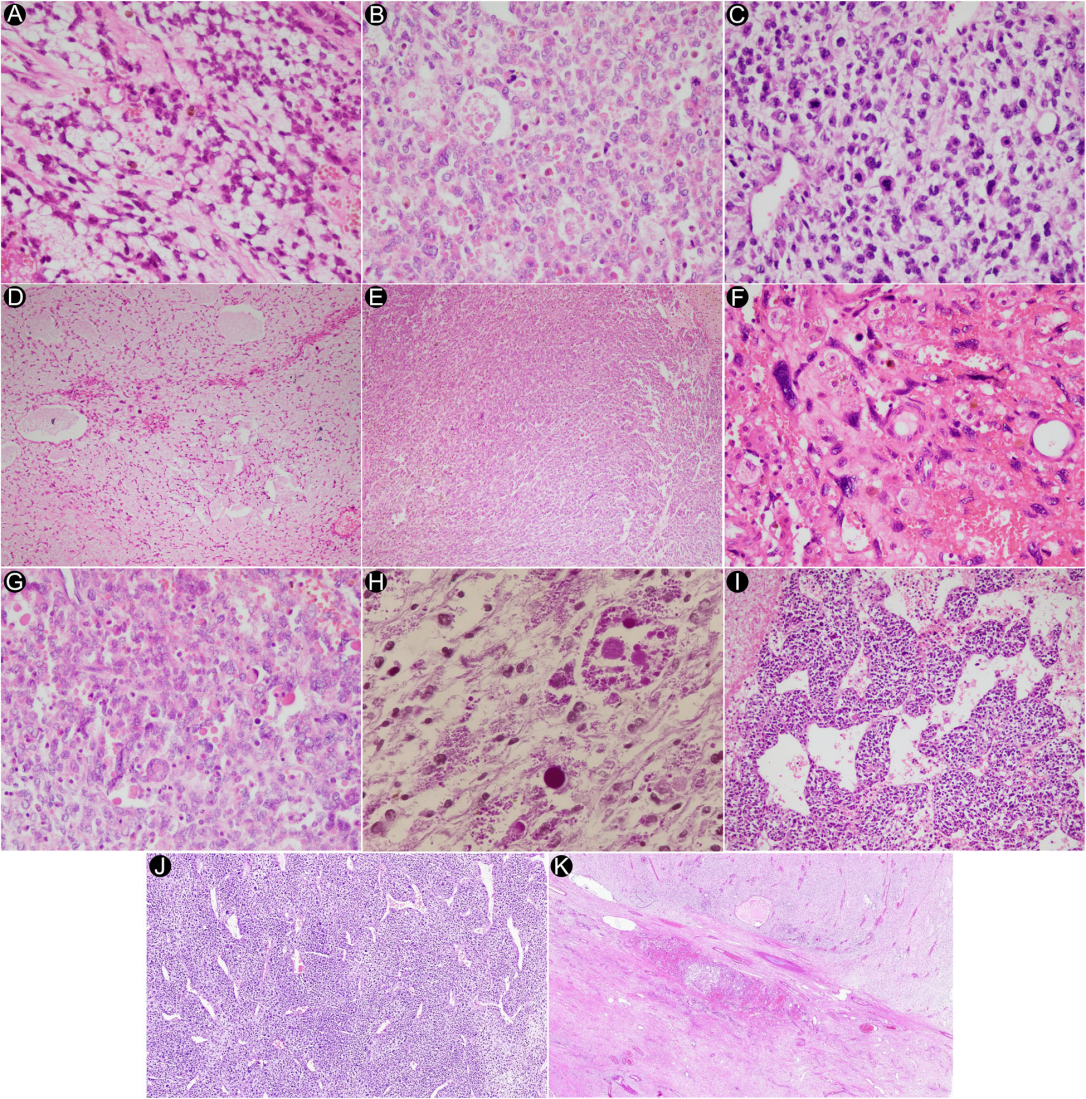
Histopathologic findings in UESL cases. **A** Anaplastic stellate and spindle-shaped tumor cells with indistinct cytoplasmic border and light cytoplasm (Hematoxylin and Eosin, 400×). **B** Anaplastic epithelioid tumor cells with eosinophilic cytoplasm (Hematoxylin and Eosin, 400×). **C** Hyperchromatic nuclei with irregular nuclear border and multiple mitoses (Hematoxylin and Eosin, 400×). **D** Loosely arranged tumoral cells (Hematoxylin and Eosin, 100×). **E** Compactly arranged tumoral cells (Hematoxylin and Eosin, 100×). **F** Bizarre and multinucleated tumor cells with highly atypical nuclei (Hematoxylin and Eosin, 400×). **G** Multiple variably-sized eosinophilic hyaline globules (Hematoxylin and Eosin, 400×). **H** Periodic acid-Schiff diastase resistant-positive hyaline globules (400×). **I** Collections of small round cells (Hematoxylin and Eosin, 200×). **J** Hemangiopericytomatous pattern (Hematoxylin and Eosin, 40×). **K** Section from liver mass showing embryonal sarcoma at the top and mesenchymal hamartoma at the bottom (Hematoxylin and Eosin, 15×)

There was no correlation between the tumor size and age at diagnosis (Spearman’s *ρ* = 0.584, *p* = 0.128). Histological findings in UESL included anaplastic stellate to spindle-shaped or epithelioid tumor cells with poorly defined, light eosinophilic cytoplasm (Fig. 1A-B). Nuclei were found to be irregular and hyperchromatic with numerous mitotic figures (Fig. 1C). The anaplastic cells were arranged loosely or compactly in a usually myxoid stroma (Fig. 1D-E). Most of the cases had bizarre multinucleated giant cells with abundant cytoplasm and atypical nuclear features (Fig. 1F). Multiple variably-sized, periodic acid-Schiff diastase resistant-positive eosinophilic hyaline globules were also frequently seen in the cytoplasm or extracellular stroma (Fig. 1G-H). Of note, collections of small round cells were also seen in two of the cases (Fig. 1I). Furthermore, hemangiopericytomatous pattern was identified in another one of the cases (Fig. 1J). Furthermore, concurrent HMH was also identified in two of UESL cases (Fig. 1K) (Table 4).

**Table 4:** Findings on histologic evaluation of UESL cases

Patient	Cellularity	Anaplasia	Spindling	Bizarre Cells	Hyaline globule (PAS stain)	Cytoplasm	Nucleoli	Small cell	Mitosis count/ Atypical	Extramedullary Hematopoiesis	Myxoid Stroma	Liver Capsule Involvement	Nontumoral liver
Case 1	Severe	+	+	+	+	Eosinophilic	Prominent	–	15/10 hpf + Atypical	–	+	–	No significant pathological change
Case 2	Moderate	+	+	+	+	Eosinophilic	Small	+	12/10 hpf + Atypical	–	+	–	No significant pathological change
Case 3	Moderate	+	+	+	+	Eosinophilic	Small	–	8/10 hpf + Atypical	–	+	–	No significant pathological change
Case 4	Severe	+	+	+	–	Eosinophilic/Clear	Large	–	20/10 hpf	–	+	–	No significant pathological change
Case 5	Moderate	+	+	+	+	Eosinophilic/Clear	Small	–	10/10 hpf + Atypical	–	+	–	Mesenchymal Hamartoma
Case 6	Moderate	+	+	+	+	Eosinophilic	Small	–	5/10 hpf	–	+	–	No significant pathological change
Case 7	Moderate	+	+	+	+	Eosinophilic	Intermediate	–	8/10 hpf + Atypical	–	+	–	No significant pathological change
Case 8	Severity	+	+	+	+	Eosinophilic/Clear	Small	+	10/10 hpf + Atypical	–	+	+	Mesenchymal Hamartoma

**Fig. 2:**
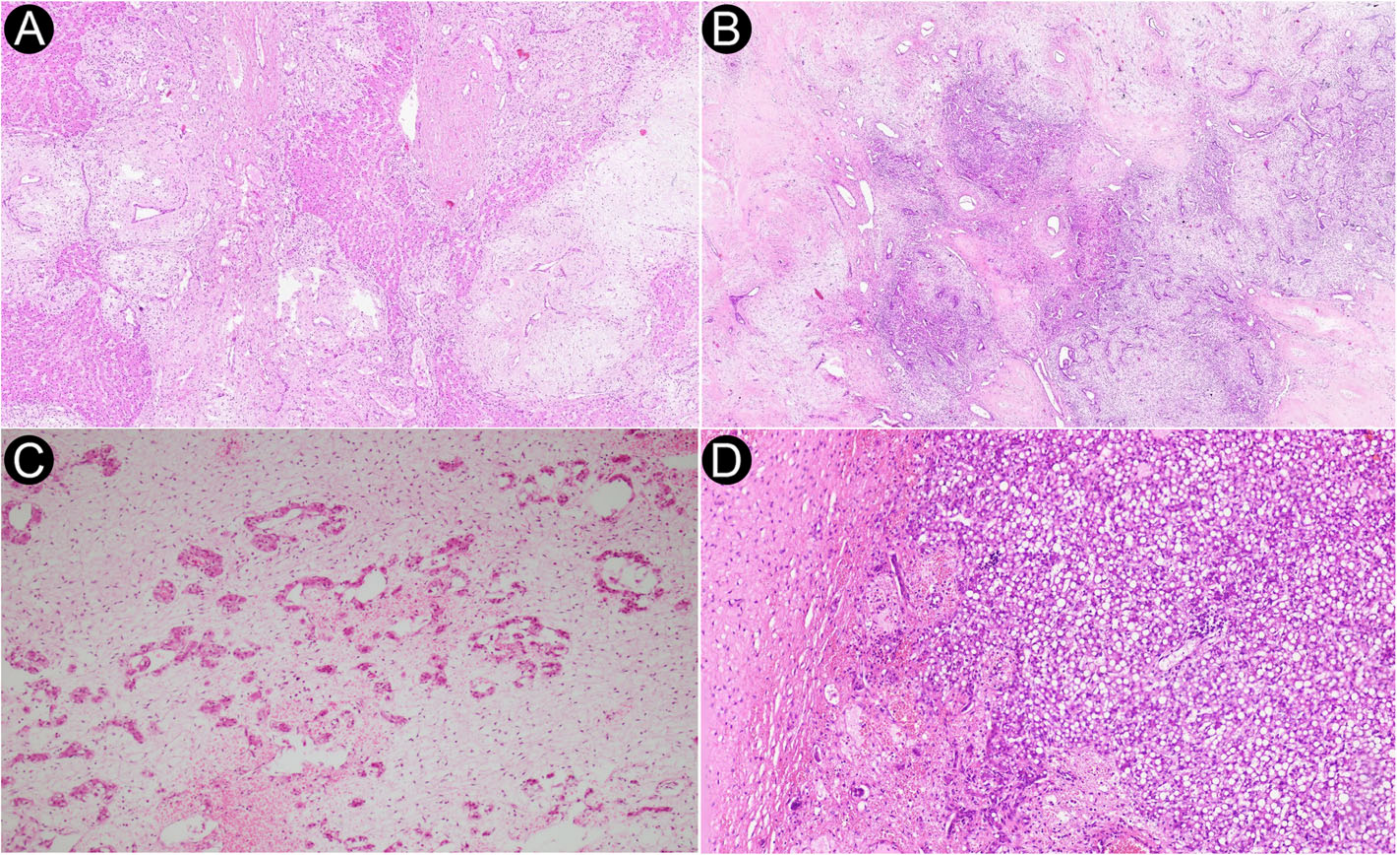
Histopathologic findings in HMH cases. **A** Disordered arrangement of hepatic parenchyma, bile ducts, and spindled cells (Hematoxylin and Eosin, 40×). **B** Disordered arrangement of hepatic parenchyma, bile ducts, and spindled cells (Hematoxylin and Eosin, 20×). **C** Hepatic parenchyma in myxoid stroma (Hematoxylin and Eosin, 100×). **D** Severe hepatic steatosis (Hematoxylin and Eosin, 80×)

**Fig. 3:**
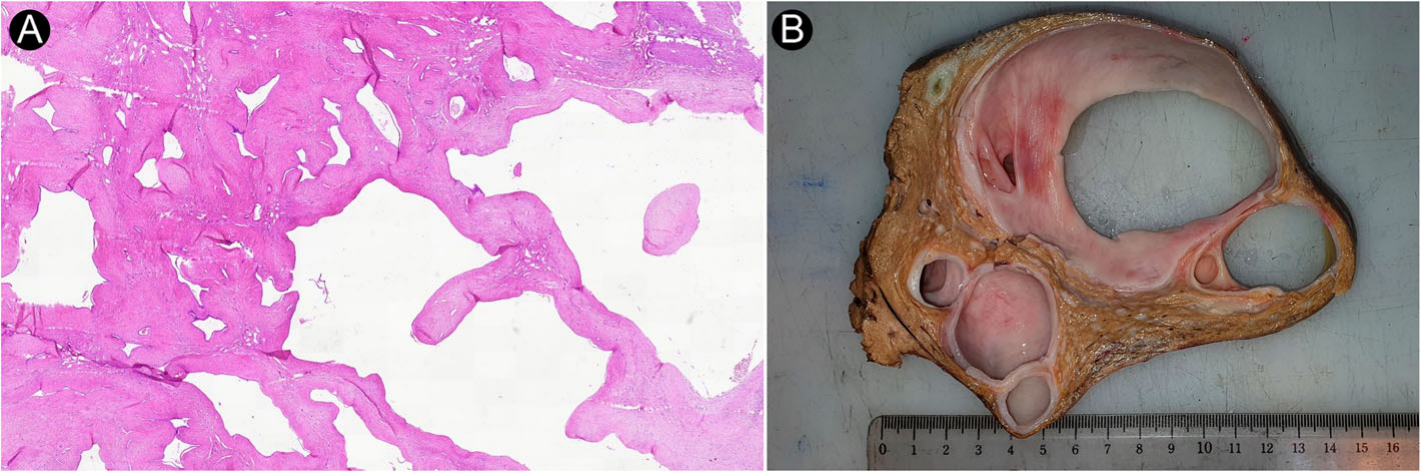
Cystic change in HMH. **A** Histopathologic section from HMH showing cystic change (Hematoxylin and Eosin, 20×). **B** Cut section of HMH with extensive cystic change

On gross evaluation, HMH cases were characterized as a well-defined solitary mass without any evidence of hemorrhage except in one of the cases. The tumor size ranged from 8.0 cm to 20.0 cm (median: 13.0 cm, IQR 11.4 cm to 15.5 cm). No correlation between the tumor size and age at diagnosis was noticed (Spearman’s *ρ* = − 0.331, *p* = 0.423). HMH was histologically characterized by the disordered arrangement of hepatic parenchyma, bile ducts, and mesenchyme consisting of spindled cells and myxoid stroma (Fig. 2A-C). Extramedullary hematopoiesis was detected in three of the cases. Furthermore, hemorrhage and severe hepatic steatosis were noted in one of the cases (Fig. 2D). Overall, 5 cases were predominantly cystic (Fig. 3A-B), while 3 cases were predominantly solid. (Table 2).

**Fig. 4:**
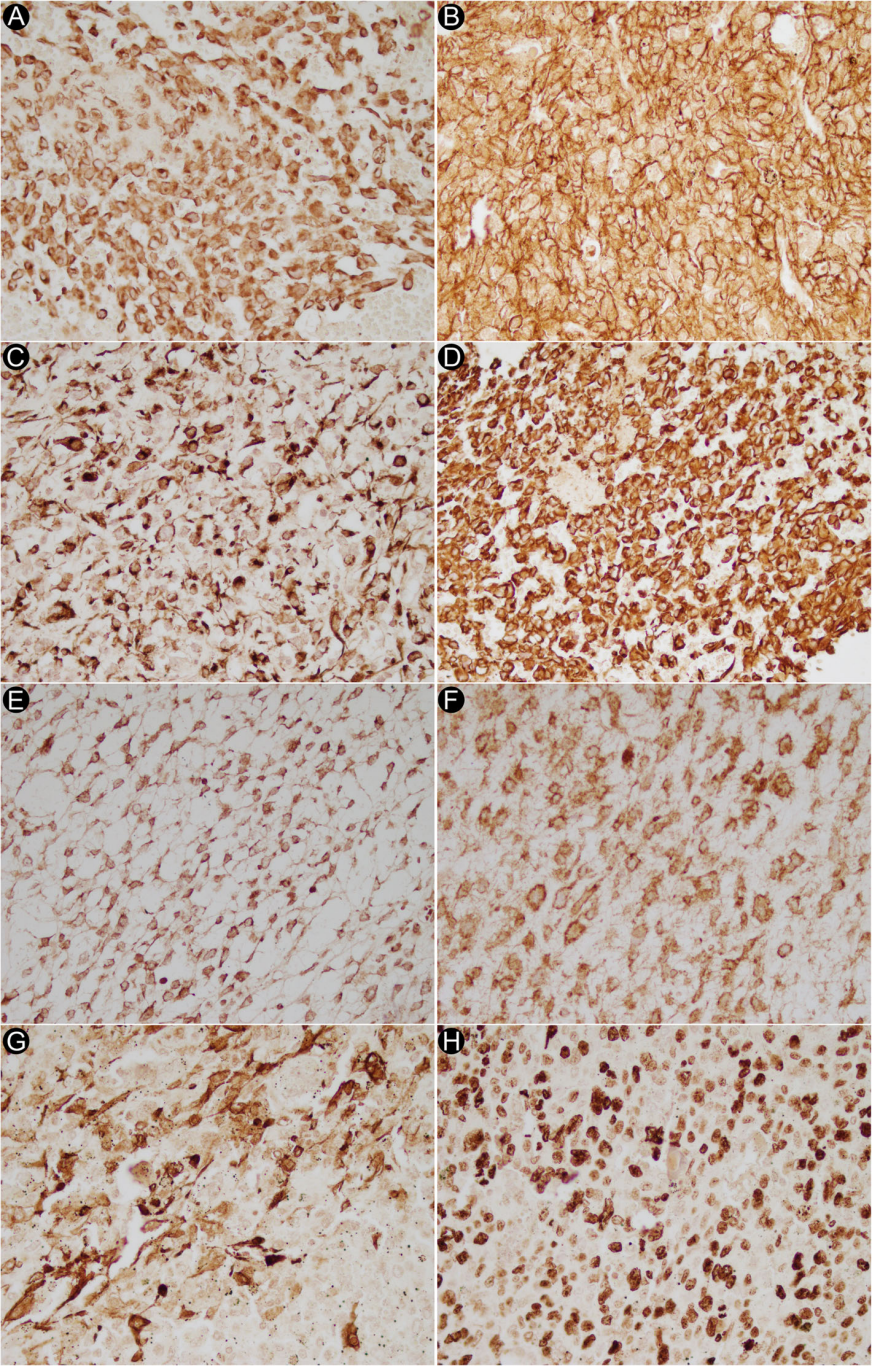
Immunohistochemical findings in UESL. (**A**) Glypican 3 (**B**) CD56 (**C**) Desmin (**D**) Vimentin (**E**) BCL2 (**F**) CD10 (**G**) SMA (**H**) Ki67 (400×)

**Fig. 5:**
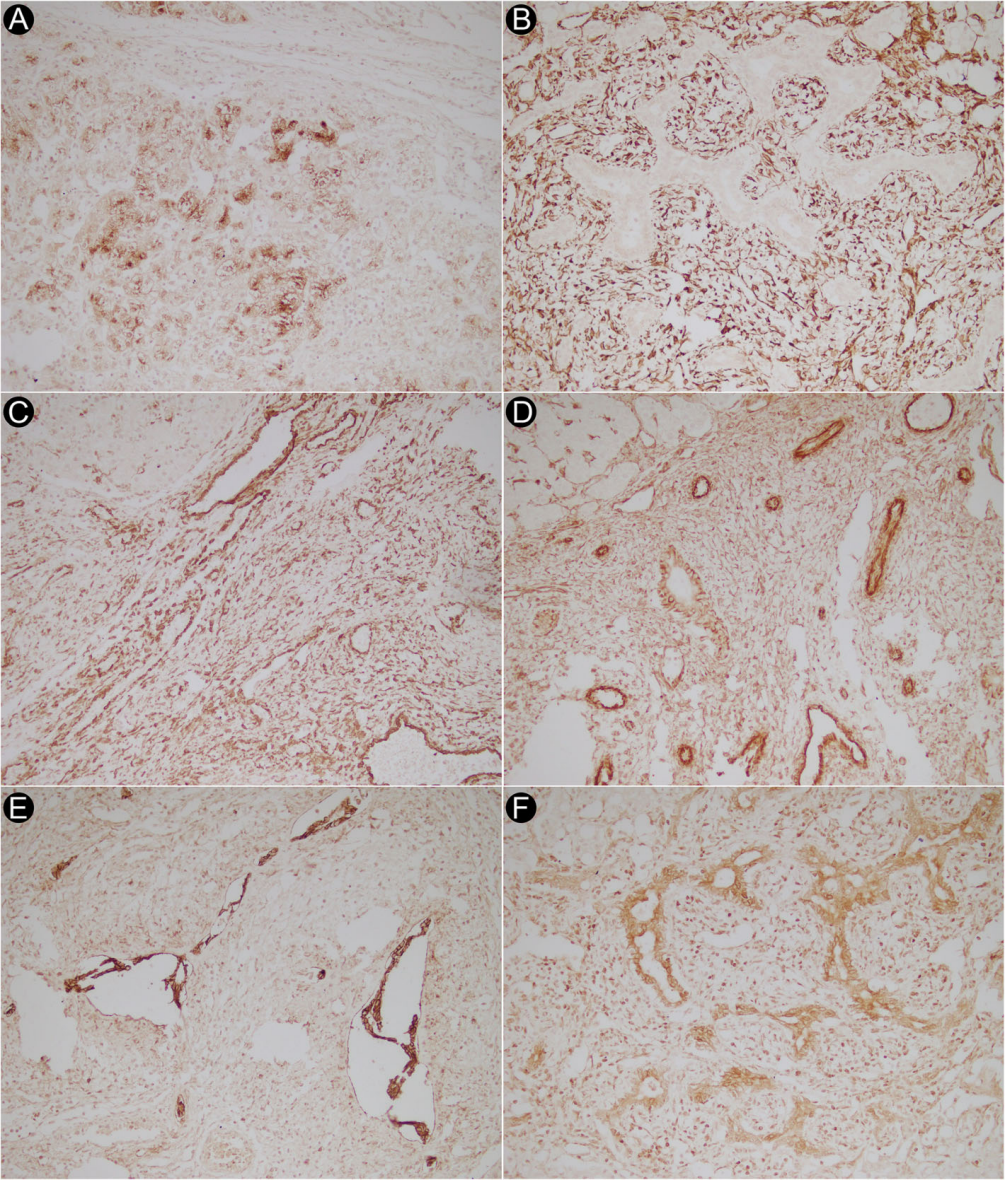
Immunohistochemical findings in HMH. (**A**) Glypican 3 (**B**) Desmin (**C**) SMA (**D**) Vimentin (**E**) CD56 (**F**) BCL2 (200×)

For immunohistochemical staining, we did not have access to the pathological specimens of two of the patients (HMH case 6 and UESL case 7) and therefore did not include them in the IHC studies. HMH associated with UESL in case 5 was included as a separate HMH case in the final analysis (Tables 5 and 6). A variable degree of Glypican 3 marker cytoplasmic staining with either strong or moderate immunoreactivity was found in UESL tumor cells. Diffuse strong cytoplasmic staining for this marker was also seen in four of HMH cases. Six UESL cases had strong cytoplasmic immunoreactivity for Desmin (3 diffuse, 3 focal) while five HMH cases showed strong cytoplasmic staining for this marker in the spindle cells (3 diffuse, 2 focal). Strong or moderate diffuse cytoplasmic staining for CD56 in all cases of UESL and also in the bile duct epithelium of HMH cases was observed. (Figs. 4 and 5).

**Table 5:** Immunohistochemistry findings in UESL cases. +++: strong staining intensity; ++: moderate staining intensity; +: weak staining intensity; f: focal staining

	Case 1	Case 2	Case 3	Case 4	Case 5	Case 6	Case 8
**Vimentin**	+++	+++f	–	+++	+++	+++	+++
**HepPar1**	–	–	–	–	–	–	–
**Glypican 3**	+++f	+++f	++	+++	+++	++f	++
**Arginase-1**	–	–	–	–	–	–	–
**Desmin**	+++	+++f	+f	+++f	+++	+++f	+++
**SMA**	–	+++f	–	+++f	+	+++f	+++f
**CD56**	+++	+++	+++	++	+++	+++	+++
**CD10**	++	+++	+++	++f	+++	–	+
**CD68**	–	–	–	+++	–	+++f	–
**BCL2**	+++	–	+	+	+	+++	+
**PD-L1**	–	–	–	–	–	–	–
**C-Kit**	–	+++f	–	–	–	–	+f
**CD34**	–	–	–	–	–	–	–
**Ki67**	80%	40%	15%	80%	60%	15%	90%

**Table 6:** Immunohistochemistry findings in HMH cases. +++: strong staining intensity; ++: moderate staining intensity; +: weak staining intensity; f: focal staining

Case #	Components	Vimentin	HepPar1	Glypican 3	Arginase-1	Desmin	SMA	CD56	CD10	CD68	BCL2	PD-L1	C-Kit	CD34	Ki67
Case 1	Spindle Cells	+++	–	–	–	+++	+++	++f	–	–	+f	–	–	–	–
	Hepatocytes	–	+++	–	++	–	–	–	–	–	–	–	–	–	–
	Bile ducts	–	–	–	–	–	–	+++	–	–	++	–	–	–	–
Case2	Spindle Cells	+++	–	–	–	+++f	+++f	+f	–	–	++f	–	–	+++	–
	Hepatocytes	–	+++	–	+++	–	–	–	–	–	–	–	–	–	–
	Bile ducts	–	–	–	–	–	–	+++	–	–	++	–	–	–	–
Case 3	Spindle Cells	+++	–	–	–	+++	+++	+++	–	–	+++	–	–	–	–
	Hepatocytes	–	++	+++	++	–	–	–	–	–	–	–	–	–	–
	Bile ducts	+++	–	–	–	–	–	+++	–	–	+++	–	–	–	–
Case 4	Spindle Cells	+++	–	–	–	+++	+++	++	–	–	++	–	–	–	–
	Hepatocytes	–	+++	+++	+++	–	–	–	–	–	–	–	–	–	–
	Bile ducts	+++	–	–	–	–	–	+++	–	–	+++	–	–	–	–
Case 5	Spindle Cells	+++	–	–	–	+++f	++	+	–	–	–	–	–	–	–
	Hepatocytes	–	+++	+++	–	–	–	–	–	–	–	–	–	–	–
	Bile ducts	–	–	–	–	–	–	+++	–	–	+++	–	–	–	–
Case 7	Spindle Cells	+++	–	–	–	–	+++	+	–	–	–	–	–	–	–
	Hepatocytes	–	+++	–	++	–	–	–	–	–	+	–	–	–	–
	Bile ducts	–	–	–	–	–	–	+++	–	–	+++	–	–	–	–
Case 8	Spindle Cells	+++	–	–	–	–	+++f	++	–	–	–	–	–	+++	–
	Hepatocytes	–	++	–	–	–	–	–	–	–	–	–	–	–	–
	Bile ducts	–	–	–	–	–	–	+++	–	–	+++	–	–	–	–
MH/UESL 5	Spindle Cells	+++	–	–	–	–	+++	–	–	–	–	–	–	+++	–
	Hepatocytes	–	+++	–	++	–	–	–	–	–	–	–	–	–	–
	Bile ducts	–	–	–	–	–	–	+++	–	–	++	–	–	–	–

## Discussion

UESL and HMH are rare hepatic lesions primarily seen in the pediatric and early adult populations. In this investigation, we, for the first time, provide a comprehensive clinicopathologic overview of these two entities in a case series from Iran. Overall, our findings show that patients with HMH are younger at presentation compared with those with UESL. Furthermore, both conditions had nonspecific initial clinical presentations with abdominal pain being present in all patients with UESL. These findings are in line with the previous investigations [6, 17]. Anemia and abnormal liver function tests were the most common findings in patients with UESL while patients with HMH had an overall normal baseline laboratory finding. Elevated tumor markers were observed in a number of patients; Elevated alpha fetoprotein levels in a HMH patient and an increased cancer antigen 125 (CA125) level was found in a patient with UESL. Elevated Erythropoietin levels have also been described in other patients with UESL which could be attributed to the mesenchymal origin of this tumor [18].

All of the patients with UESL had undergone gross surgical resection of the tumor followed by chemotherapy except for one of the patients who had not received chemotherapy. In addition, two of the patients with UESL also received radiotherapy. In the past, the prognosis of liver sarcomas was poor overall. In the original report of 31 patients with UESL by Stocker and Ishak, only six patients were alive without any evidence of the disease [7]. Over the past couple of years, mounting evidence suggests that radical surgical resection of the tumor supplemented by adjuvant chemotherapy leads to an overall improvement in the survival of these patients [19]. Herein, we report encouraging results of following this protocol in patients with UESL. Six out of eight patients had no evidence of disease after a median follow-up time of 3 years. We were only able to retrieve the data regarding the chemotherapy regimen for three of the patients. However, based on our limited findings, all three of the patients who showed no evidence of disease on the latest follow up received vincristine and cyclophosphamide along with other chemotherapeutic agents. Treatment with vincristine, actinomycin-D, cyclophosphamide has been shown to be a successful therapeutic strategy in another report [20]. Notably, one of the patients had no evidence of disease 6 years after the diagnosis only by gross tumor resection. This in turn highlights the paramount importance of surgical tumor resection in the treatment of this disorder.

On gross evaluation, all of the masses were located in the right hepatic lobe with variable sizes of up to 28 cm. UESL masses usually showed hemorrhage and cystic changes with variable degrees of necrosis while HMH cases presented with unifocal solid or cystic structures. Small round cells were seen on histologic evaluation of two UESL cases which could be an important finding if present in liver biopsies from these patients and can mimic other pathologies. In addition, severe hepatic steatosis seen in one of HMH cases points to potential molecular defects leading to deregulation of cellular energetics as seen in other liver disorders [21]. Furthermore, UESL was diagnosed in concurrence with HMH in two of the studied patients. This finding which has been also reported in a previous case series points to the potential malignant transformation of HMH in such patients [15].

The immunohistochemical phenotypes of UESL and HMH have only been investigated in a few studies so far. Overall, in three case series, positive immunostaining for vimentin and Bcl-2 has been reported in most of the cases while positive staining for desmin, SMA, p53, pancytokeratin, Glypican-3, and calponin has been observed in some of the cases. Immunostaining for HepPar1, CD34, CD117 (C-kit), S100, HMB45, myogenin, ALK-1, and α-fetoprotein were found to be negative in the primary tumor cells [22–24]. In addition, positive immunostaining for desmin, vimentin, SMA, Glypican-3, Hep Par 1, and α-fetoprotein in different components of HMH has been reported [24–26]. Our investigation highlighting immune-reactivity for CD56 in UESL and different components of HMH expands the findings of previous case series studies and corroborates their findings for other markers as well. Furthermore, our findings demonstrating the expression of Bcl-2 and CD34 in various components of HMH not only broadens its immunophenotypic spectrum but also provides fresh impetus for further investigations regarding the malignant transformation of HMH since both these two markers showed moderate to strong immunoreactivity in different components of the HMH case found in association with UESL. The development of UESL after incomplete excision of HMH reported in the literature corroborates this hypothesis [27, 28].

Absence of PD-L1 expression in all of the UESL cases investigated in this study points to the potential lack of efficacy of immune checkpoint inhibitors targeting this pathway as a therapeutic target in these patients [29]. However, positive staining for CD56 which is also reported in rhabdomyosarcoma and synovial sarcoma could have significant therapeutic implications in the management of UESL [30, 31]. CD56–chimeric antigen receptor T-cell therapy which has already shown promising results in the pre-clinical studies in other cancers could be used in the treatment of patients with a high CD56 expression in the tumor tissue who had shown disease recurrence with poor response to chemotherapy and radiotherapy (e.g. UESL case 6 in this study) [32].

The main differential diagnoses of UESL in the pediatric and adolescent population include hepatoblastoma, HMH, embryonal rhabdomyosarcoma, hepatic angiosarcoma, and hepatocellular carcinoma [23, 33]. IHC staining could play a substantial role in accurate diagnosis particularly in situations where histopathologic clues are not helpful. Our study showing Glypican-3 expression in both UESL and HMH cases demonstrates that this marker cannot be used to differentiate the two from hepatocellular carcinoma and hepatoblastoma which have been shown to express this antigen [34, 35]. Nevertheless, our study showed absence of HepPar 1 and Arginase-1 in all of the UESL cases highlighting the importance of this maker in differentiating UESL from hepatocellular carcinoma for which these two markers have been shown to display a sensitivity of 84.4 and 96.0%, respectively [36]. Positive staining for skeletal muscle differentiation markers such as myoD1 and myogenin along with cross-striation are distinctive findings in rhabdomyosarcoma [37]. Furthermore, CD34 marker which was found to be negative in all of the cases in this investigation can be a very useful marker in the diagnosis of hepatic angiosarcoma which frequently stains positive for this marker [38]. Features such as anaplasia and high grade mitotic activity (strongly positive staining for ki-67) as observed in this study are useful diagnostic features to distinguish UESL from HMH.

In summary, this investigation reports the first case series of patients with UESL and HMH from Iran. Herein, we reported the clinicopathologic findings of sixteen patients from a single referral pediatric center. Although due to the rarity of these two pathologic entities we were not able to recruit a large number of patients in order to investigate the prognostic significance of different pathologic findings, we were able to identify significant histopathologic findings and novel IHC markers with diagnostic and therapeutic implications. Further investigations have to be conducted to shed light on the clinicopathologic and pathophysiologic basis of these two rare entities.

## Data Availability

All data generated or analyzed during this study are included in this published article.

## Abbreviations

UESL: Undifferentiated embryonal sarcoma of the liver
HMH: Hepatic mesenchymal hamartoma
PAS: Periodic Acid–Schiff
IQR: Interquartile range
